# Long-Term Outcomes of Antegrade Continence Enemas to Treat Constipation and Fecal Incontinence in Children

**DOI:** 10.1097/MPG.0000000000003833

**Published:** 2023-05-17

**Authors:** Desiree F. Baaleman, Mana H. Vriesman, Peter L. Lu, Marc A. Benninga, Marc A. Levitt, Richard J. Wood, Desale Yacob, Carlo Di Lorenzo, Ilan J.N. Koppen

**Affiliations:** From the * Division of Gastroenterology, Hepatology, and Nutrition, Nationwide Children’s Hospital, Columbus, OH; the † Department of Pediatric Gastroenterology and Nutrition, Emma Children’s Hospital, Amsterdam UMC, University of Amsterdam & VU University, Amsterdam, The Netherlands; the ‡ Center for Colorectal and Pelvic Reconstruction, Nationwide Children’s Hospital, Columbus, OH.

**Keywords:** appendicostomy, cecostomy, child, constipation, fecal incontinence

## Abstract

The objective of this study is to investigate long-term outcomes of antegrade continence enema (ACE) treatment in children with constipation or fecal incontinence.

**Methods:**

Prospective cohort study including pediatric patients with organic or functional defecation disorders who started ACE treatment. Data were collected at baseline and at follow-up (FU) from 6 weeks until 60 months. We assessed parent and patient-reported gastrointestinal health-related quality of life (HRQoL) using the Pediatric Quality of Life Inventory Gastrointestinal Symptoms Module (PedsQL-GI), gastrointestinal symptoms, adverse events, and patient satisfaction.

**Results:**

Thirty-eight children were included (61% male, median age 7.7 years, interquartile range 5.5–12.2). Twenty-two children (58%) were diagnosed with functional constipation (FC), 10 (26%) with an anorectal malformation, and 6 (16%) with Hirschsprung disease. FU questionnaires were completed by 22 children (58%) at 6 months, 16 children (42%) at 12 months, 20 children (53%) at 24 months, and 10 children (26%) at 36 months. PedsQL-GI scores improved overall with a significant increase at 12- and 24-month FU for children with FC and a significant increase in parent reported PedsQL-GI score at 36-month FU for children with organic causes. Minor adverse events, such as granulation tissue, were reported in one-third of children, and 10% of children needed a surgical revision of their ACE. The majority of all parents and children reported that they would “probably” or “definitely” choose ACE again.

**Conclusion:**

ACE treatment is perceived positively by patients and parents and can lead to long-term improvement in gastrointestinal HRQoL in children with organic or functional defecation disorders.

## Graphical abstract

**Figure:**
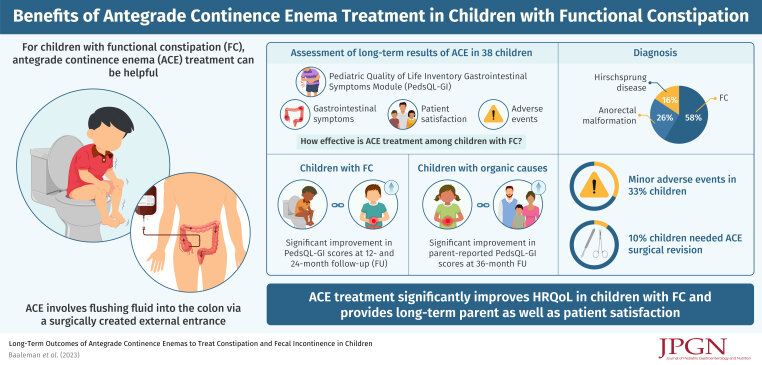

**What Is Known**

- Retrospective studies or studies with a short-term follow-up have shown that antegrade continence enema (ACE) treatment can be effective in treating children with intractable constipation or fecal incontinence.

**What Is New**

- ACE treatment can lead to long-term improvement in gastrointestinal health-related quality of life in children with organic and functional constipation.
- Child and parent satisfaction rates were especially high in children with organic constipation.
- Minor adverse events were reported in approximately one-third of children, and 10% of children needed a surgical revision of their ACE.

Antegrade continence enema (ACE) treatment can be helpful for children with functional constipation (FC) or organic causes of constipation refractory to conventional medical treatment (1,2). The ACE procedure entails flushing fluid into the colon via a surgically created external entrance into the colon, at the appendix for a Malone appendicostomy and in the cecum for a cecostomy (1). The irrigation fluid consists of tap water or saline to which stimulant laxatives may be added to increase colonic peristalsis. During the Malone procedure, a surgeon connects the appendix to the abdominal wall to create an intestinal conduit using an open or laparoscopic surgical approach. In patients lacking a usable appendix the surgeon may tubularize a cecal flap to establish the same result (3). The percutaneous approach involves a minimally invasive alternative with placement of a cecostomy tube or “button” device through the abdominal wall. The tube can be placed using an image-guided technique by interventional radiologists, endoscopically, or surgically using an open or laparoscopic approach. The clinical efficacy of both procedures is considered similar but reported complication rates vary (1,4). In some cases, these procedures are combined with the resection of a colonic segment if it is extremely dilated or shows signs of severe dysmotility. Previous studies investigating the clinical efficacy of the ACE procedure are mostly retrospective case series. One prospective study describes outcomes of ACE in a cohort of 80 children with FC. In this study, the majority of patients (70%) report satisfactory control of their symptoms without a clear definition of the meaning of satisfactory control. The long-term clinical effectiveness of ACE in children with defecation disorders, as well as the duration of treatment, and the effect of ACE treatment on health-related quality of life (HRQoL) remain unclear. Therefore, the objective of our study was to prospectively evaluate HRQoL and clinical outcomes of children starting ACE treatment at our institution.

## METHODS

A prospective cohort study was conducted in the GI Motility Center and the Center for Colorectal and Pelvic Reconstruction at Nationwide Children’s Hospital in Columbus, OH. The local Institutional Review Board approved the study (STUDY00000497). We included children with FC or organic causes of constipation initiating ACE treatment at our institution from 2015 to 2019. During the patients’ admission for the ACE procedure, caregivers and children were invited to participate in the study. Participants were excluded if they had limited English language proficiency. All caregivers gave written consent, and children aged 9 years and above gave assent. After enrollment, we collected demographic information, medical history, surgical history, procedural information, and information on symptom severity. Parents and children completed questionnaires at baseline, and at follow-up (FU) at 6 weeks (range 3–9 weeks), 6 months (range 3–9), 12 months (range 9–15), 24 months (range 16–30), 36 months (range 30–42), 48 months (range 42–54), and 60 months (range 54–66).

### HRQoL

The Pediatric Quality of Life Inventory Gastrointestinal Symptoms Module (PedsQL-GI) was used to assess perceptions of gastrointestinal HRQoL. The scale is comprised of parallel parent proxy-report and child self-report formats. Both formats are comprised of subscales, including a constipation-specific symptom scale. Items are reverse-scored and linearly transformed to a 0–100 scale (0 = 100, 1 = 75, 2 = 50, 3 = 25, 4 = 0); higher scores indicate fewer problems or symptoms and, hence, a higher HRQoL. The total score and subscale scores are computed as the sum of the items divided by the number of items answered. In addition, parents completed the Parental Opinions of Pediatric Constipation Questionnaire. The questionnaire consists of 24 items scored on a 5-point Likert scale divided in 4 subscales: burden/distress, family conflict, difficulties with the medical team, and worry about social impact. Higher scores represent more issues, and hence, a lower HRQoL. Data are presented separately for children with FC and children with organic causes of constipation and compared with PedsQL-GI data of another study reporting data of healthy American children. In addition, in accordance with the core outcome set of children with FC, we evaluated how many days of school children missed (5).

### Clinical Outcomes and Adverse Events

We collected data on clinical symptoms and adverse events at each FU. A self-developed questionnaire was used to evaluate patient experience and satisfaction with the ACE and adverse social implications. Patients no longer using ACE were asked about the reason for cessation of treatment.

### Statistical Analysis

Data were analyzed with the use of SPSS version 21.0 (SPSS Institute, Chicago, IL). Because of our small sample size, we assumed data were not normally distributed. Therefore, data are presented using medians and interquartile ranges. Some participants did not complete all questionnaires, and missing data were excluded from analyses. To avoid type I errors, only our main outcomes (PedsQL-GI and weekly fecal incontinence) were statistically tested comparing FU to baseline using McNemar test, or Wilcoxon Signed Rank test as appropriate. Data analysis was performed following a modified intention-to-treat principle, including all children of whom data was available, regardless of their ACE use. A *P* value less than 0.05 was considered statistically significant.

## RESULTS

We included 38 patients who started treatment with ACE [61% male, median age 7.7 years, interquartile range (IQR) 5.5–12.2], see Table 1. Twenty-two children (58%) were diagnosed with FC, 10 (26%) were diagnosed with an anorectal malformation, and 6 (16%) were diagnosed with Hirschsprung disease. All children with organic disorders had undergone surgical correction before ACE was considered. Children with Hirschsprung disease had undergone colonic resection at the level of the sigmoid (n = 5) or rectum (n = 1). Of all patients, most (n = 24; 63%) were using rectal enemas or saline irrigations at baseline, some (n = 6; 16%) used these rectal treatments in combination with oral laxatives. Twelve patients (32%) were only using oral laxatives at baseline, and 2 patients (anorectal malformation: n = 1; FC: n = 1) were not using any medication for their constipation or fecal incontinence. Thirteen patients (33%) started ACE treatment despite doing well with rectal enemas (no fecal incontinence and a weekly defecation frequency of 3 or more bowel movements per week). The majority of these children (n = 12) wanted to start ACE in order to stop treatment with rectal enemas. Reasons to discontinue rectal enemas included: parental preference (n = 5); uncooperativeness with enema administration (n = 3); prolonged duration of rectal enema administration (n = 1); liquid stool long after rectal enema administration (n = 1); to increase quality of life (n = 1); or to increase independence (n = 1). The other patient wanted to start ACE because the child still had hard and big bowel movements despite a bowel movement frequency of 3 per week. Twenty-nine children (76%) underwent a laparoscopic Malone appendicostomy, 4 (11%) a percutaneous cecostomy, 3 (8%) an open Malone appendicostomy, and 2 (5%) a Malone appendicostomy combined with a sigmoid resection. Figure Supplemental Digital Content 1, http://links.lww.com/MPG/D178 depicts the FU patient flowchart. FU questionnaires were completed by 19 children (50%) at 6 weeks, 22 children (58%) at 6 months, 16 children (42%) at 12 months, 20 children (53%) at 24 months, 10 children (26%) at 36 months, 8 children (21%) at 42 months, and 6 children (16%) at 60 months.

**TABLE 1.** Baseline characteristics

	Functional constipation (n = 22)	Anorectal malformation (n = 10)	Hirschsprung disease (n = 6)
Male, n (%)	12 (55%)	5 (50%)	6 (100%)
Age in years, median (IQR)	9.7 (6.0–13.1)	5.7 (4.0–7.7)	6.4 (4.8–9.2)
**Comorbidities**			
ADHD, n (%)	4 (18%)	0 (0%)	0 (0%)
Autism spectrum disorder, n (%)	5 (23%)	0 (0%)	0 (0%)
Cerebral palsy, n (%)	1 (4%)	0 (0%)	0 (0%)
Tethered cord, n (%)	0 (0%)	3 (30%)	0 (0%)
Trisomy 21, n (%)	0 (0%)	0 (0%)	2 (33%)
VACTERL association, n (%)	0 (0%)	4 (40%)	0 (0%)
**Diagnostic test results**			
Contrast enema, N	22	7	6
Normal/as expected, n (%)	5 (23%)	3 (43%)	5 (83%)
Dilated (recto)sigmoid, n (%)	10 (45%)	1 (14%)	0 (0%)
Redundant (recto)sigmoid, n (%)	8 (36%)	2 (29%)	1 (17%)
Redundant transverse colon, n (%)	0 (0%)	1 (14%)	0 (0%)
Focal stricture, n (%)	1 (5%)	0 (0%)	0 (0%)
Anorectal manometry, N	18	0	3
Normal, n (%)	10 (56%)		0 (0%)
Pelvic floor dyssynergia, n (%)	5 (28%)		0 (0%)
Increased resting pressure, n (%)	1 (6%)		0 (0%)
Inconclusive, n (%)	2 (11%)		0 (0%)
Absent recto-anal inhibitory reflex, n (%)	1 (6%)*		3 (100%)
Colonic manometry, N	17	0	1
Normal, n (%)	10 (59%)		1 (100%)
Distal segmental dysmotility, n (%)	6 (35%)		0 (0%)
Minimal motility: 1 HAPC in 2 days, n (%)	1 (6%)		0 (0%)
**Surgical procedure**			
Laparoscopic Malone, n (%)	16 (73%)	7 (70%)	5 (83%)
Percutaneous cecostomy, n (%)	3 (14%)	0 (0%)	1 (17%)
Classic Malone, n (%)	1 (5%)	2 (20%)	0 (0%)
Malone and sigmoid resection, n (%)	2 (9%)	0 (0%)	0 (0%)
Laparoscopic Malone and Redo PSARP, n (%)	n/a	1 (5%)	n/a

ADHD = attention deficit hyperactivity disorder; HAPC = high amplitude propagated contraction; IQR = interquartile range; PSARP = posterior sagittal anorectoplasty.

* The rectal biopsy of this child showed normal ganglion cells.

### HRQoL

**FIGURE 1.**
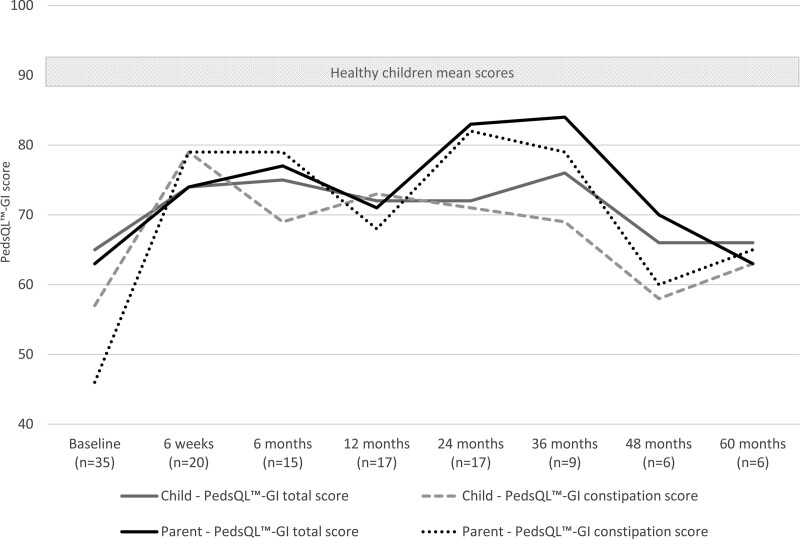
Health-related gastrointestinal quality of life over the course of the study of all participants. PedsQL-GI = Pediatric Quality of Life Inventory Gastrointestinal Symptoms Module.

As shown in Figure 1 and Table Supplemental Digital Content 2, http://links.lww.com/MPG/D179, there was an overall increase in both parent- and child-reported PedsQL-GI scores with a significant increase for children with FC at 12- and 24-month FU and for children with organic causes of constipation in parental reported GI total score at 36-month FU. As shown in Table Supplemental Digital Content 2, http://links.lww.com/MPG/D179, Parental Opinions of Pediatric Constipation Questionnaire scores and number of missed schooldays showed an overall decrease at each FU point compared to baseline.

### ACE Use

The majority of children continued to use their ACE during the FU period, see Table 2. At 6-week FU, 1 child with FC discontinued ACE because the tube fell out. At 6-month FU 1 child with FC discontinued ACE because the “child neglected to use the ACE.” The same child was reported not to use the ACE at 12- and 24-month FU because “he let the button grow over,” and “the problem resolved,” respectively. At 36-month FU, 1 child with an organic cause of constipation discontinued ACE because they wanted to try oral medications again, and 1 child with FC discontinued ACE because it was no longer needed. At 48-month FU, 2 children with FC discontinued ACE, one because “he had his tube removed” and the other had a colostomy placed because treatment with ACE was not deemed beneficial. The last child also reported at 60-month FU that they did not use their ACE because it “stopped working.” To summarize, at least 3 children with FC were able to wean ACE treatment because of resolution of symptoms and 1 child with FC had a colostomy placed because ACE treatment was not effective.

**TABLE 2.** Outcomes of antegrade continence enema treatment at baseline and follow-up

	Baseline	6 weeks	6 months	12 months	24 months	36 months	48 months	60 months
**Clinical outcomes**								
ACE continued use								
Functional constipation, n/N (%)	n/a	6/7 (86%)	12/13 (92%)	8/9 (89%)	8/9 (89%)	3/4 (75%)	2/4 (50%)	1/2 (50%)
Organic disorders, n/N (%)	n/a	11/11 (100%)	8/8 (100%)	6/6 (100%)	7/7 (100%)	5/6 (83%)	2/2 (100%)	3/3 (100%)
ACE flush frequency								
Functional constipation, median (IQR)	n/a	7 (7–7)	7 (7–7)	7 (7–8.5)	7 (6–7.5)	7 (5–7)	7 (7–8)	7
Organic disorders, median (IQR)	n/a	7 (7–7)	7 (7–7)	7 (7–7)	7 (7–7)	7 (7–7)	3.5 (2–3.5)	7 (3–7)
Fecal incontinence								
Functional constipation, n/N (%)	14/22 (64%)	1/7 (14%)	2/13 (15%)	1/9 (11%)	1/9 (11%)	1/4 (25%)	1/4 (25%)	2/3 (67%)
Organic disorders, n/N (%)	9/16 (56%)	3/11 (27%)	3/8 (38%)	3/6 (50%)	3/7 (43%)	2/5 (40%)	1/3 (33%)	2/3 (67%)
Hard stools								
Functional constipation, n/N (%)	11/21 (52%)	1/7 (14%)	1/13 (8%)	0/9 (0%)	3/9 (33%)	0/4 (0%)	1/4 (25%)	1/3 (33%)
Organic disorders, n/N (%)	2/10 (20%)	1/11 (9%)	1/8 (13%)	1/6 (17%)	0/7 (0%)	0/6 (0%)	1/3 (33%)	1/3 (33%)
Abdominal pain								
Functional constipation, n/N (%)	16/22 (73%)	5/7 (71%)	6/13 (46%)	3/9 (33%)	4/9 (44%)	2/4 (50%)	2/4 (50%)	3/3 (100%)
Organic disorders, n/N (%)	4/11 (36%)	2/11 (18%)	2/8 (25%)	3/6 (50%)	3/7 (43%)	1/6 (17%)	2/3 (67%)	2/4 (67%)
**Patient satisfaction**								
Parental satisfaction on scale from 0 to 10*								
Functional constipation, median (IQR)	n/a	6.8 (3.2–9.7)	8.7 (5.2–10)	9.7 (6.5–10)	9.0 (5.0–9.9)	7.0 (0–7.0)	5.0 (0–5.0)	5.0 (0.5–5.0)
Organic disorders, median (IQR)	n/a	9.8 (8.8–10)	9.7 (9.0–10)	8.7 (7.2–9.2)	9.1 (8.6–9.9)	8.8 (8.3–9.9)	10 (7.4–10)	9.1 (9.0–9.1)
Parental satisfaction: would probably or definitely choose ACE again†								
Functional constipation, n/N (%)	n/a	3/5 (60%)	9/10 (90%)	9/9 (100%)	10/11 (91%)	3/3 (100%)	2/3 (67%)	2/3 (67%)
Organic disorders, n/N (%)	n/a	12/12 (100%)	8/8 (100%)	7/7 (100%)	8/8 (100%)	6/6 (100%)	3/3 (100%)	3/3 (100%)
Child satisfaction on scale from 0 to 10*								
Functional constipation, median (IQR)	n/a	6.9 (0.8–6.9)	6.6 (4.4–9.2)	7.6 (5.2–9.6)	5.1 (3.7–10)	9.3 (6.0–9.3)	5.0 (0.0–5.0)	2.7 (0.0–2.7)
Organic disorders, median (IQR)	n/a	9.6 (7.1–10)	9.7 (5.3–9.7)	7.9 (5.0–7.9)	9.2 (7.7–9.8)	8.9 (5.7–9.9)	9.5 (6.9–9.5)	8.5 (7.3–8.5)
Child satisfaction: would probably or definitely choose ACE again‡								
Functional constipation, n/N (%)	n/a	1/3 (33%)	4/5 (80%)	4/8 (50%)	4/7 (57%)	2/3 (67%)	1/3 (33%)	0/3 (0%)
Organic disorders, n/N (%)	n/a	4/5 (80%)	2/3 (67%)	3/3 (100%)	4/4 (100%)	4/4 (100%)	2/3 (67%)	2/2 (100%)

ACE = antegrade continence enema; IQR = interquartile range.

* On a Likert-scale from 0 to 10; higher scores represent higher levels of satisfaction.

† On a scale from never, possible, maybe, probably, or definitely.

‡ On a scale from never, possible, maybe, probably, or definitely, only completed by children from 8 years of age.

Over the course of the study, children reported that the time from start of the flush until completion of defecation took on average a median of 45 minutes (IQR 45–60). When evaluating all FU data of the study, at 48 of 74 (65%) measurements children reported to use stimulants into their ACE flush. There was no difference in children experiencing pain or discomfort during flushing between those who did or did not use stimulants [15/48 (31%) with stimulants and 8/26 (31%) without stimulants]. In addition, there was no difference in how often children reported complete evacuation after flushing between those who did or did not use stimulants. At 30 of 82 (36%) measurements children reported to stool in between flushes; this varied from 19%–50% at each FU point without a clear increase or decrease over time.

### Clinical Outcomes

**FIGURE 2.**
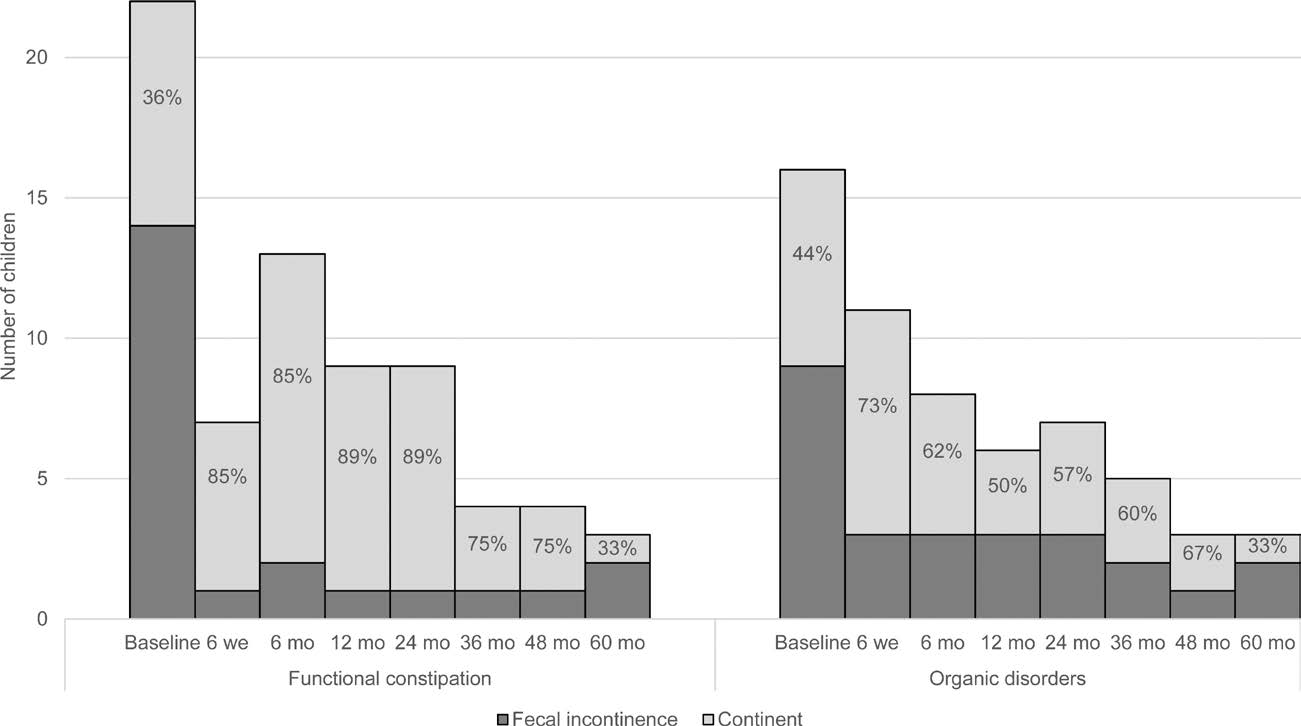
Fecal incontinence rates at baseline and follow-up.

A general decrease in gastrointestinal symptoms was observed. As shown in Figure 2 and Table 2, we found no statistically significant improvement in the presence of fecal incontinence at FU compared to baseline. Fecal incontinence rates of children with FC at 6-month and 24-month FU almost reached significance compared to baseline, with *P* values of 0.07 and 0.063, respectively.

### Patient Satisfaction

Parent and child ACE satisfaction scores at each FU are shown in Table 2. For children with FC, median ACE satisfaction rates among children and their parents ranged from 2.7 to 9.7 (on a scale from 0 = not at all satisfied to 10 = extremely satisfied). The majority of parents reported that they would “probably” or “definitely” choose ACE again (60%–100%), and a varying rate of children reported that they would “probably” or “definitely” choose ACE again (0%–80%). For children with organic causes of constipation, median ACE satisfaction rates among both children and their parents were high (all median scores above 8.5) at each FU. At each FU, all parents and the majority of children (67%–100%) with organic causes of constipation reported that they would “probably” or “definitely” choose ACE again.

### Adverse Events

Adverse events were common, with approximately one-third of children reporting at least 1 adverse event (Table Supplemental Digital Content 3, http://links.lww.com/MPG/D180). Because of low numbers, differences in complication rates between children with organic or functional defecation disorders were not statistically tested. The majority of children whom reported complications were diagnosed with FC. One of the most reported adverse events was granulation tissue at the Malone or cecostomy site, and was usually treated with silver nitrate applications. Three children required a surgical revision of the ACE due to closure, prolapse, and a switch from cecostomy to appendicostomy due to overlying bowel loops. One child had a stenosis which required a surgical dilation, and another child ended up with a colostomy at the 48-month FU. Social implications of ACE treatment were apparent at the start but decreased over time. At 6-week FU, around a third of children felt ashamed for their ACE, tried to hide their ACE, and were hindered by social activities according to their parents.

## DISCUSSION

This is the first long-term prospective evaluation of children with severe constipation or fecal incontinence treated with ACE. ACE treatment led to improvement in gastrointestinal HRQoL, including an overall improvement in gastrointestinal symptoms but without a significant decrease in fecal incontinence at FU visits compared to baseline. Child and parent satisfaction rates were especially high in children with organic causes of constipation. Minor adverse events were reported in approximately one-third of children, and 10% of children needed a surgical revision of their ACE.

For children with FC, we found a significant increase in HRQoL at 12- and 24-month FU. This is in line with a previous prospective study in 15 patients with FC reporting an increase in HRQoL measured via the PedsQL generic core at 6- and 12-month FU compared to baseline (6). However, the results of that study should be interpreted with caution given that they used 2-tailed Student paired *t* test to test for significant differences but with the small sample size of 15 children it is unlikely that these data were normally distributed. In our study, parents were more positive about choosing ACE and reported higher satisfaction scores compared to children. This may be secondary to adverse social effects of ACE treatment experienced by children, with a proportion of children reporting that they tried to hide their ACE or felt ashamed of their ACE.

For children with organic causes of constipation, we found a significant increase in HRQoL at the 36-month FU. No other studies have prospectively evaluated the effect of ACE on HRQoL in children with anorectal malformations or Hirschsprung disease. Studies in children with anorectal malformations report success rates of ACE treatment of 72%–96% (7–9). A previous study in children with Hirschsprung disease reported a success rate of ACE treatment, defined as normal stooling pattern with no reported soiling, of 50% (7). Another study including a heterogeneous group of children reported that a history of Hirschsprung disease was associated with poorer outcome of ACE treatment (10). Authors of that study described that poorer outcome in children with Hirschsprung disease may be secondary to abnormal motility patterns in the remaining ganglionic bowel and previous surgical treatment.

Although the clinical benefit of ACE was more prominent in children with FC with an increase in HRQoL and decrease of fecal incontinence rates, children and parents with organic causes of constipation seemed more satisfied with their ACE. Differences in patient satisfaction between children with FC and organic causes of constipation and their parents may arise from differences in their prior experience with other (surgical) treatment and comorbidities. Most children with organic diseases have undergone surgery before and may suffer from other invalidating symptoms requiring invasive treatment. This may result in them considering the ACE treatment as relatively less invasive compared to children with FC and their parents in which ACE may have been their only surgical intervention and treatment.

One cross-sectional study compared the effect of ACE treatment on stool symptoms and HRQoL in 34 children with FC and organic causes of constipation. Among the 21 who completed the questionnaires, the authors found that the beneficial effect of ACE treatment was similar in both groups. However, they did not use validated questionnaires, and more importantly, they collected the data on stool symptoms and HRQoL based on recall at a median of 3.5 years after ACE placement, asking participants in retrospect about symptoms and HRQoL preoperatively, 3 months postoperatively, and at latest FU. This introduces both participation and recall bias and makes it difficult to compare these data with our prospective data. Other studies report conflicting findings, some reporting equal or higher failure rates in children with FC, while others, as noted before, report an association between poor outcome and a history of Hirschsprung disease (7,10,11). These differences may be secondary to differences in patient selection, work-up, and prior treatment before initiation of ACE. The highest levels of treatment success are described in a study among 348 children with anorectal malformations in which the authors describe a detailed work-up, patient selection, and individualized treatment regime (12). This underlines the need for more research to evaluate which patients respond better to certain treatments. Research to date shows that in children with FC, dyssynergic defecation does not seem to predict ACE response (13). Abnormal colonic manometry results have been associated with the necessity of surgical treatment, but prospective studies using colonic manometry to guide treatment have yet to be performed (14). Besides improving patient selection and outcomes, guidelines should be developed for the testing and surgical treatment of children with intractable FC, and for children with postoperative obstructive defecation symptoms with organic diseases.

Adverse events, such as granulation tissue, were common in approximately a third of children in our cohort, with 10% of children requiring surgical revision of the site. This is in line with a literature review including 3976 children, among whom nearly one-third developed similar complications and approximately 20% required surgical revision (2). This study reported that when long-term FU was taken into consideration, the complication rate actually rose to 95.2%. The review did not find evidence for differences in complication rates based on underlying disorder. In our cohort, most children who reported complications were diagnosed with FC.

Strengths of our study include the prospective design, the use of validated questionnaires to measure HRQoL, and long-term FU. We decided to analyze results of children with FC and organic causes of constipation separately, improving the reliability and generalizability of our results. However, this resulted in small sample sizes putting our study at risk to be underpowered which impaired our ability to identify statistical significant differences. One of our main limitations includes the high rate of loss to FU, especially after 36 months. The limited number of completed questionnaires may have resulted in selection bias, which could be responsible for the lower quality of life scores found at 5-year FU. Our longest FU data therefore have to be interpreted with caution. Another limitation includes the use of limited inclusion criteria with many children already fulfilling criteria for treatment success while using other treatments, especially in the FC group. In the FC group, many children were diagnosed with psychological comorbidities, which may limit the generalizability of our findings. Children with behavioral problems may be less receptive to rectal treatment, which may lead them to consider ACE to reduce stress accompanied with rectal enema treatment. Therefore, a randomized controlled trial comparing the efficacy, HRQoL, side effects, independence, and patient/parental satisfaction of rectal enema or transanal irrigation treatment to ACE would be of great interest. In addition, qualitative research is needed to explore why some children tolerate rectal treatment with a good effect on their symptoms, while others do not tolerate rectal treatment at all.

## CONCLUSIONS

To conclude, ACE treatment led to improvement in HRQoL, particularly in children with FC, and a decrease in fecal incontinence, although not statistically significant. Adverse events were common with 1 in 10 patients requiring surgical revision of the Malone or cecostomy site. This should be clearly discussed with parents when considering an ACE—especially in children who are doing well with rectal enema treatment. Patient and parent satisfaction were high after several years of treatment. ACE can be considered as treatment option for children with constipation unresponsive to conventional medical treatment before more invasive surgical treatment is initiated.

## Supplementary Material


