# Supplementary figures and images for: Long-Term Outcomes of Antegrade Continence Enemas to Treat Constipation and Fecal Incontinence in Children
Source: J Pediatr Gastroenterol Nutr. 2023 May 17;77(2):191–7. doi: 10.1097/MPG.0000000000003833 (PMC10348609; doi:10.1097/MPG.0000000000003833)

Figure, Supplemental Digital Content 1. Patient flow chart

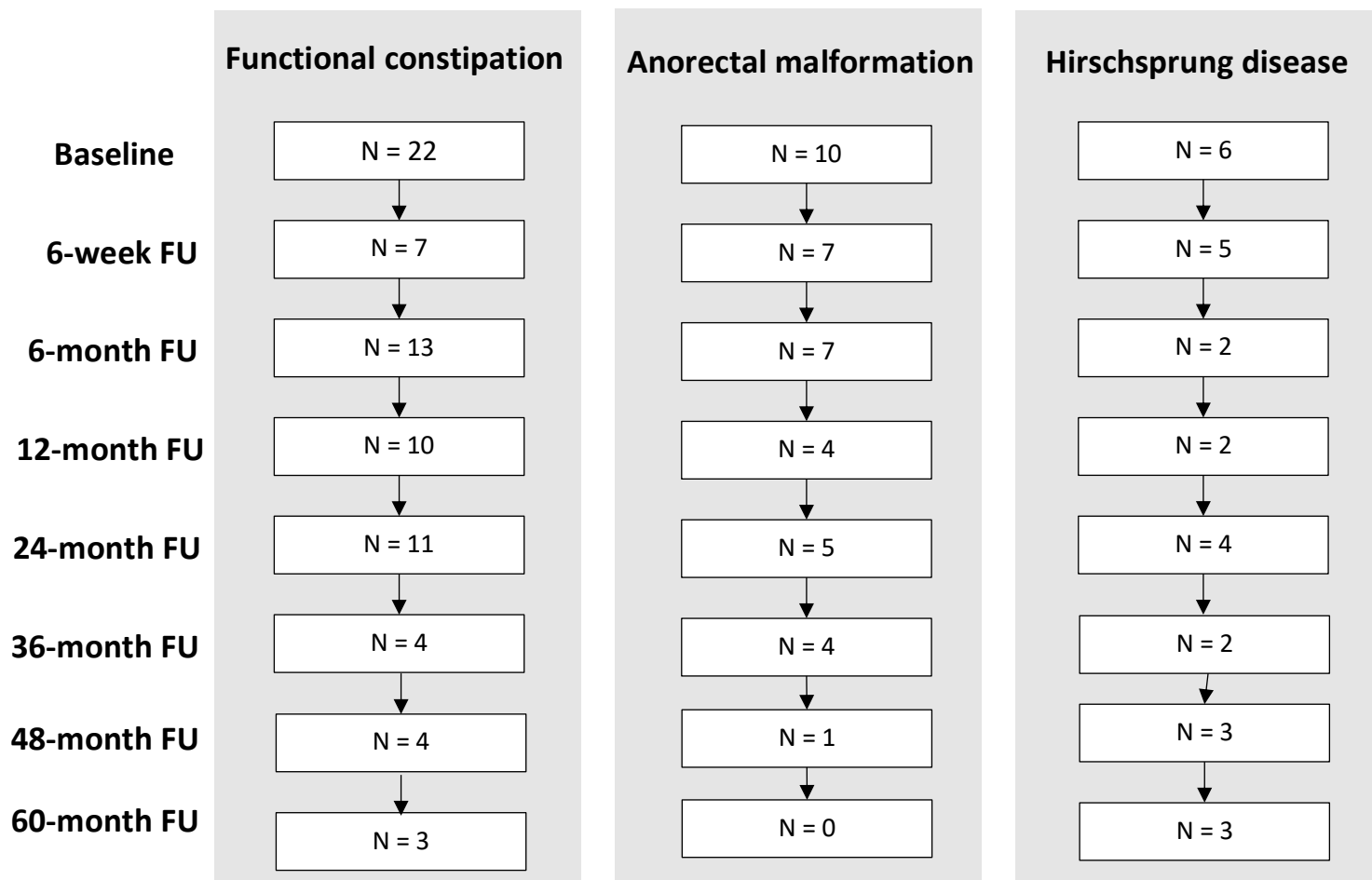

Supplement: Supplementary file 1 [file mpg-77-191-s001.pdf]
