# Supplementary material for: Long-Term Outcomes of Antegrade Continence Enemas to Treat Constipation and Fecal Incontinence in Children
Source: J Pediatr Gastroenterol Nutr. 2023 May 17;77(2):191–7. doi: 10.1097/MPG.0000000000003833 (PMC10348609; doi:10.1097/MPG.0000000000003833)
Supplement: Supplementary file 2 [file mpg-77-191-s002.pdf]

Table, Supplemental Digital Content 2. Health-related quality of life and missed schooldays from baseline to follow-up

	**Baseline** (35/38)	**6 weeks** (n = 17)	**6 months** (n = 18)	**12 months** (n = 15)	**24 months** n = 18	**36 months** N = 9	**48 months** N = 6	**60 months** N = 6
Child - PedsQL™-GI total score, [a]								
- FC, median (IQR)	62 (48-78)	67 (53-73)	79 (52-92)	74 (63-95)*	74 (61-82)*	61 (45-61)	61 (45-61)	60 (36-60)
- Organic, median (IQR)	75 (59-90)	78 (68-94)	75 (67-90)	76 (60-91)	70 (52-84)	77 (64-83)	77 (64-83)	No data
Child - PedsQL™-GI constipation score, [a]								
- FC, median (IQR)	49 (24-83)	59 (46-74)	64 (25-88)	79 (60-93)*	75 (46-94)*	69 (66-69)	63 (21-63)	55 (23-55)
- Organic, median (IQR)	64 (50-88)	88 (62-100)	78 (63-100)	71 (46-94)	64 (38-86)	68 (58-88)	54 (48-54)	No data
Parent - PedsQL™-GI total score, [a]								
- FC, median (IQR)	58 (47-68)	69 (61-70)	64 (52-76)	69 (54-82)	78 (63-90)**	62 (38-62)	64 (30-64)	63 (25-63)
- Organic, median (IQR)	80 (62-86)	78 (74-91)	84 (78-90)	83 (59-92)	83 (65-91)	87 (67-96)*	55 (29-55)	68 (59-68)
Parent - PedsQL™-GI constipation score, [a]								
- FC, median (IQR)	45 (17-63)	61 (54-78)	57 (37-73)	64 (52-74)	91 (59-95)**	66 (55-66)	64 (30-64)	63 (25-63)
- Organic, median (IQR)	70 (33-84)	92 (72-96)	92 (79-100)	82 (54-96)	80 (57-93)	87 (67-96)	55 (29-55)	68 (59-68)
POOP-C total score, [b]								
- FC, median (IQR)	72 (60-76)	65 (48-71)	64 (48-79)	52 (48-70)	47 (38-63)	66 (40-66)	67 (48-67)	53 (48-53)
- Organic, median (IQR)	58 (52-72)	51 (43-59)	45 (31-56)	52 (35-56)	52 (36-64)	45 (28-60)	68 (52-74)	58 (33-58)
POOP-C burden/worry score, [b]								
- FC, median (IQR)	32 (30-34)	28 (24-30)	25 (21-34)	23 (19-29)	22 (11-31)	28 (12-28)	32 (31-33)	25 (22-25)
- Organic, median (IQR)	28 (24-31)	20 (17-34)	17 (9-19)	24 (15-29)	22 (14-26)	19 (8-24)	29 (21-31)	25 (17-25)
Number of missed schooldays last month, median (IQR) [c]	3.5 (0-9.8)	1.0 (0-3.0)	0 (0-2.0)	0 (0-2.0)	0 (0-2.5)	0 (0-0)	0 (0-1.0)	0 (0-0)

PedsQL™-GI, Pediatric Quality of Life Inventory Gastrointestinal Symptoms Module; *POOPC,* Parental Opinions of Pediatric Constipation Questionnaire

[a] Higher scores represent higher levels of health-related quality of life, scores range from 0 to 100.

Child- PedsQL™-GI N = 28, 13, 12, 13, 12, 8, 6, 4 respectively.

Parent- PedsQL™-GI total score N = 35, 20, 15, 17, 17, 9, 6, 6, respectively.

Parent- PedsQL™-GI constipation N = 35, 20, 15, 16, 17, 9, 6, 6, respectively.

[b] Higher scores represent more worries/concerns, burden/worry score ranges from 8-40; N = 35, 22, 15, 18, 18, 9, 7, 6, respectively.
total score ranges from 24-120; N = N = 35, 22, 15, 18, 18, 9, 6, 6, respectively.

[c] excluding children who were on summer holiday at time of questionnaire completion

* depicts P<0.05 compared to baseline; ** depicts P<0.01 compared to baseline; *** depicts P< 0.001 compared to baseline
