# Supplementary material for: Long-Term Outcomes of Antegrade Continence Enemas to Treat Constipation and Fecal Incontinence in Children
Source: J Pediatr Gastroenterol Nutr. 2023 May 17;77(2):191–7. doi: 10.1097/MPG.0000000000003833 (PMC10348609; doi:10.1097/MPG.0000000000003833)
Supplement: Supplementary file 3 [file mpg-77-191-s003.pdf]

Table, Supplemental Digital Content 3. Adverse events and social effects of antegrade continence enema (ACE) treatment at follow-up

	6 weeks	6 months	12 months	24 months	36 months	48 months	60 months
**New complication reported, n/N (%)**	**6/18 (33%)**	**6/21 (29%)**	**3/15 (20%)**	**4/16 (25%)**	**0/10 (0%)**	**1/7 (14%)**	**0/6 (0%)**
**Cumulative percentage, n/N (%)**	**6/18 (33%)**	**10/26 (39%)**	**11/27 (41%)**	**13/30 (43%)**	**13/32 (41%)**	**13/34 (38%)**	**13/34 (38%)**
- Surgical site infection, [a] n (%)	-	1 (5%)	-	-	-	1 (13%)	-
- Pain during ACE flush, [b] n (%)	2 (10%)	-	1 (6%)	2 (10%)	-	1 (13%)	-
- Granulation tissue, [c] n (%)	3 (15%)	3 (14%)	1 (6%)	1 (5%)	-	1 (13%)	-
- Stoma leakage, [d] n (%)	3 (15%)	1 (5%)	-	-	-	1 (13%)	-
- Other, n (%)	2 (10%)	3 (14%)	-	3 (15%)	-	-	-
**Required treatment**							
- Colostomy, n (%)	-	-	-	-	-	1 (13%)	-
- Malone surgical revision, n (%)	-	2 (10%)	1 (6%)	-	-	-	-
- Surgical dilation, n (%)	-	1 (5%)	-	-	-	-	-
- Tube change, n (%)	1 (5%)	1 (5%)	1 (6%)	1 (5%)	-	-	-
- Botox injection, n (%)	-	-	1 (6%)	-	-	-	-
- Antibiotics, n (%)	1 (5%)	1 (6%)	1 (6%)	2 (10%)	-	-	-
- Silver nitrate, n (%)	3 (15%)	1 (6%)	-	-	-	-	-
**Social effects of ACE treatment**							
Child – total N	9	7	11	11	7	6	5
I am ashamed for ACE, [e] n (%)	3 (33%)	3 (43%)	2 (18%)	1 (9%)	0 (%)	0 (0%)	2 (40%)
I try to hide my ACE, [e] n (%)	4 (44%)	4 (57%)	5 (46%)	2 (18%)	2 (29%)	1 (17%)	1 (20%)
I am afraid other children do not like me because of my ACE, [e] n (%)	1 (11%)	1 (14%)	0 (0%)	0 (0%)	0 (0%)	1 (17%)	2 (40%)
I avoid activities where people see my ACE, [e] n (%)	1 (11%)	1 (14%)	3 (27%)	0 (0%)	1 (14%)	1 (17%)	1 (20%)
Parent – total N	20	15	17	24	9	6	6
Child is hindered in social activities, [f] n (%)	9 (45%)	4 (27%)	3 (18%)	2 (11%)	3 (33%)	2 (33%)	3 (50%)

[a] Of patients who reported complication; two patients diagnosed with functional constipation

[b] Of patients who reported complication; three patients diagnosed with functional constipation; three with Hirschsprung disease

[c] Of patients who reported complication; seven patients diagnosed with functional constipation; two with Hirschsprung disease

[d] Of patients who reported complication; four patients diagnosed with functional constipation.

[e] Completed by children from 8 years of age, scored on a 5-point Likert scale from 1 (strongly disagree) to 5 (strongly agree): includes children who agreed or strongly agreed

[f] Scored on a 5-point Likert scale from 1 (not al all) to 5 (always): includes parents who answered: sometimes, often, or always
